# Influence of a multi-strain probiotic and zinc-glycine chelate, administered *in ovo*, on immune response in newly hatched chicks

**DOI:** 10.3389/fphys.2025.1646143

**Published:** 2025-09-22

**Authors:** Artur Ciszewski, Łukasz S. Jarosz, Zbigniew Grądzki, Agnieszka Marek, Beata Kaczmarek, Marcin Hejdysz, Anna Rysiak

**Affiliations:** ^1^ Department of Epizootiology and Clinic of Infectious Diseases, Faculty of Veterinary Medicine, University of Life Sciences in Lublin, Lublin, Poland; ^2^ Department of Preventive Veterinary and Avian Diseases, Faculty of Veterinary Medicine, University of Life Sciences in Lublin, Lublin, Poland; ^3^ Department and Clinic of Animal Internal Diseases, Sub-Department of Internal Diseases of Farm Animals and Horses, Faculty of Veterinary Medicine, University of Life Sciences in Lublin, Lublin, Poland; ^4^ Department Of Animal Breeding And Product Quality Assessment, Poznań University of Life Sciences, Poznań, Poland; ^5^ Department of Botany, Mycology, and Ecology, Maria Curie-Skłodowska University, Lublin, Poland

**Keywords:** *in ovo*, chelated zinc, multi-strain probiotic, cytokines, acute-phase proteins, immunoglobulins

## Abstract

**Introduction:**

The supplementation of chicken embryos with bioactive compounds may elicit a beneficial effect on the development of their gut microbiome and enhance protection against infectious agents after hatching. Therefore, this study aimed to evaluate the effect of *in ovo* co-supplementation with a multi-strain probiotic and zinc-glycine chelate on the levels of pro- and anti-inflammatory cytokines, acute-phase proteins, and immunoglobulins in the peripheral blood and tissues of broiler chickens on the day of hatching and 7 days post hatching. The effect of supplementation on the growth parameters of chickens was assessed as well.

**Methods:**

The study was conducted on 1,500 hatching eggs from a broiler breeding flock (Ross × Ross 308) at 36 weeks. ELISA kits were used to determine levels of acute-phase proteins and immunoglobulins. Expression of immunoglobulins was determined by means of qRT-PCR.

**Results:**

The results indicate enhanced synthesis of acute-phase proteins in the liver and increased levels of serum amyloid A in the small intestine tissue, as well as IgA and IgM mRNA and suppressed synthesis of pro-inflammatory cytokines IFN-γ and TNF-α. During the cumulative experimental period (days 0–42), the mean body weight gain (BWG) and feed intake (FI) in the group supplemented with a multi-strain probiotic were statistically significantly lower than the control group.

**Discussion:**

It may be concluded that the combined in ovo use of a multi-strain probiotic and Zn-Gly chelate modulates the immune response, helps maintain the balance between the synthesis of Th1 and Th2 cytokines, inhibits inflammatory processes, and stimulates immune system development.

## 1 Introduction

The growing demand for poultry products has contributed to the growth of poultry production and necessitated the development of new technologies for rearing chickens with favorable nutritional and dietary qualities (Mottet and Tempio, 2017; Dong et al., 2024). A critical time in poultry rearing is the peri- and post-hatching period, which significantly influences the survival rate of chicks and their weight gain (Almeida et al., 2006). One recently promoted method for improving the health of chicks entails the *in ovo* administration of bioactive compounds to the embryo (Kpodo and Proszkowiec-Weglarz, 2023). This technique enables the formation of a beneficial profile of the intestinal microbiome at the embryo stage, reduces the extent of oxidative stress, enhances protection against infectious agents by increasing immunization efficiency, and increases the energy reserves of the embryo and chick (Arain et al., 2022; Ayalew et al., 2023). A subject of particular interest is the use of multi-strain probiotics and organic forms of zinc, which when administered *in ovo* enhance the immune system of chickens by stimulating the production of antibodies (Zulkifli et al., 2000; Kabir et al., 2004; Haghighi et al., 2006), T lymphocytes, and the pro-inflammatory cytokines IFN-γ, IL-12, and IL-1β, which in turn stimulate Th1 immune response and help increase the mass of lymphoid organs (Incharoen et al., 2019; Zhang et al., 2021; Rousseaux et al., 2023). Similarly, the administration of Zn in organic form in the post-hatch period promotes the growth, differentiation, and activation of immune cells, including macrophages, B, and T cells (mainly TCD4+), and enhances phagocytosis (Jarosz et al., 2017a). It also promotes the synthesis and secretion of antibodies, increases the antioxidative potential of cells (Huang et al., 2007; Moghaddam and Jahanian, 2009; Sahoo et al., 2014), and inhibits IL-1β and IL-6 production, thereby reducing inflammation in the cells (Jin et al., 2023).

An even more beneficial effect on the immune system of birds can be achieved by combining multi-strain probiotics and organic forms of zinc *in ovo* (Ciszewski et al., 2023a). This administration strategy promotes mRNA expression of cytokines in the cecal tonsils, bursa of Fabricius, and spleen in the post-hatch period (Alizadeh et al., 2020). Similarly, *in ovo* administration of Zn stimulates cellular immune mechanisms in the post-hatch period and increases mRNA expression of a zinc exporter in the small intestine and of brush border enzymes and transporter genes (Tako et al., 2005). *In ovo* administration of probiotics also ensures the early multiplication of beneficial bacteria in the intestines, leading to changes in the intestinal microbiome (Schokker et al., 2015). As a consequence of these modifications, the microbes contained in the probiotic positively influence immunocompetent cells of the intestinal epithelium and modulate cellular and humoral mechanisms of the immune response, helping reduce bacterial infections in chickens in the early stage of life (de Oliveira et al., 2014; Schokker et al., 2015). However, these interactions can also upset homeostasis in the intestines and lead to inflammation. Similarly, excessive *in ovo* administration of highly bioavailable zinc in chelated form can cause reduced hatching rates in poultry (Kim and Kang, 2022), inflammatory lesions, erosion in the gut, and changes in the biodiversity of the intestinal microbiome (Starke, et al., 2014). Data from the literature indicate that alongside its beneficial effects, the *in ovo* administration of both substances can induce stress in the embryo, leading to inflammation and acute-phase response, which is one of the components of the innate immune response (Ciszewski et al., 2023b). This leads to the synthesis of acute-phase proteins (APP) in the liver, which upon release to the bloodstream become markers of ongoing inflammation (Gruys et al., 2005). The development of acute-phase response (APR) is accompanied by a decrease in the concentration of zinc in the fluids and tissues, which is redistributed from the blood—mainly to the liver (Liuzzi et al., 2006).

The mechanisms involved in the immune response in poultry following the application of multi-strain probiotics together with zinc-glycine (Zn-Gly) chelates are not well understood. We therefore hypothesized that *in ovo* supplementation with a multi-strain probiotic and Zn-Gly chelate may modulate the early immune response in broiler chickens by influencing cellular and humoral mechanisms, including the synthesis of immunoregulatory proteins involved in maintaining the Th1/Th2 balance. The aim of this study was to determine the impact of the *in ovo* co-supplementation of a multi-strain probiotic and Zn-Gly chelate on the concentrations of pro- and anti-inflammatory cytokines, acute-phase proteins, and immunoglobulins in the peripheral blood and tissues of broiler chickens on the day of hatching (DOH) and at 7 days post-hatch, as well as on the weights of the lymphatic organs. Additional analyses were conducted to assess the expression of mRNA immunoglobulin in the small intestine and the effect of these preparations on the growth parameters of chickens throughout the rearing period. The results of this study will help elucidate the role of multi-strain probiotics and Zn-Gly chelate in immune processes in chickens in the first few days of their life.

## 2 Materials and methods

### 2.1 Experimental animals and ethics committee approval

The research material comprised 1,500 hatching eggs obtained from a commercial flock of broiler breeders at the age of 36 weeks (Ross × Ross 308) delivered to the Experimental Station at the Poznań University of Life Sciences (ul. Gorzyń 4, Międzychód commune, Poland). All procedures used in the research were approved by the Local Ethics Committee for Animal Testing at the University of Life Sciences in Lublin, Poland (approval no. 106/2022 of 17 October 2022).

### 2.2 Incubation of eggs and *in ovo* supplementation with the analyzed preparations

The eggs (1,500) were stored at 21 °C under commercial conditions for 24 h before being placed in the incubation chamber. They were randomly distributed and incubated in JARSON Model JD-18 incubators (Gostyń, Poland) at the Experimental Station of the Department of Animal Nutrition and Feed Management Gorzyń/Miedzychód (Poland). For the first 18 days, the eggs were incubated under standard commercial conditions: 37.8 °C and relative humidity (RH) 55%–60%. On the 19th day of incubation (DOI), they were transferred to a JARSON Model ATLAS-180 hatching chamber (Gostyń, Poland). From days 19–21, the hatching parameters were 37.7–38.1 °C and RH 60%–65%. At 7 and 17 DOI, all eggs were candled and cracked, and unfertilized and dead embryonic eggs were removed.

On embryonic day 17, 1,400 fertilized eggs were randomly distributed into four treatment groups, with 10 replicates per group and 35 eggs per replicate (350 eggs per group). They were assigned to groups according to the preparation administered *in ovo* as follows: eggs injected with sterile 0.9% physiological saline (control group–I); eggs injected with a multi-strain probiotic–group II (1 × 10^5^ CFU/egg *Saccharomyces cerevisiae*; 1 × 10^7^ CFU/egg *Lacticaseibacillus casei*; 1 × 10^7^ CFU/egg *Lactiplantibacillus plantarum*); eggs injected with a multi-strain probiotic and Zn-Gly chelate– group III (1 × 10^5^ CFU/egg *S. cerevisiae*; 1 × 10^7^ CFU/egg *L. casei*; 1 × 10^7^ CFU/egg *L. plantarum*; 100 µg/egg Zn-Gly); eggs injected with Zn-Gly – group IV (100 µg/egg Zn-Gly). The eggs from each group received an equal volume (500 μL) of 0.9% physiological saline or a bioactive compound, injected *in ovo* into the amniotic sac. The *in ovo* injection procedure has been described in detail by Alizadeh et al. (2020), Alizadeh et al. (2021), Ciszewski et al. (2023a), and Ciszewski et al. (2023b).

### 2.3 Bioactive compounds

The multi-strain probiotic EM Provet used in the experiment was manufactured by Greenland Technologia EM (Janowiec, Poland); its exact composition has been described by Ciszewski et al. (2023a). EM Provet was provided in powdered form and diluted in PBS (phosphate-buffered saline) to obtain a solution containing 1 × 10^5^ CFU *S. cerevisiae*, 1 × 10^7^ CFU *L. casei*, and 1 × 10^7^ CFU *L. plantarum* in a 100 µL of solution. The Zn-Gly chelate used in the experiment was manufactured by Arkop Sp. z o.o. (Bukowno, Poland). The Zn-Gly powder contained 250 mg of Zn-Gly per g of product. The powder was dissolved in deionized water to obtain solutions containing 100 µg of Zn-Gly/100 µL MQ water. In experimental groups III and IV, 100 µg of Zn-Gly/100 µL MQ water was administered to each egg, corresponding to 30.33 µg of elemental Zn per egg. All procedures are described in detail by Ciszewski et al. (2023a) and Ciszewski et al. (2023b).

### 2.4 Animals and feeding program

After hatching, 150 one-day-old male chicks from each group were selected at random for analysis. Each treatment group had 10 replicates with 15 birds per replicate. The 42-day growing trial was conducted at the Experimental Station of the Department of Animal Nutrition and Feed Management, Gorzyń/Miedzychód (Poland). The experiment was performed in fully controlled environmental conditions and in accordance with the recommendations for this line (Aviagen, Ross Broiler Management Handbook, Ross 308/308 FF Broiler: Performance Objectives; Aviagen, Ross Broiler Management Handbook). The birds were reared on wood shavings in pens measuring 1.2 × 0.8 m. The pens were equipped with feeding lines and nipple drinkers. For the first 3 days, the temperature in the experimental room at the experimental station was maintained at 33 °C ± 1 °C and was then successively lowered by 3 °C per week until reaching 24 °C ± 1 °C. The lighting program was 24 h of light for the first 10 days and then 19 h of light and 5 h of darkness. Relative humidity (RH) was maintained at 60% ± 10% throughout the experimental period. Water and feed were provided *ad libitum* throughout the entire experimental period. The birds were fed a basal diet with no coccidiostats or antibiotics. The feed was provided in crumbled form, prepared at the Experimental Station at the Poznań University of Life Sciences in Gorzyń (Poland) using a SKIOLD SK2500 disc mill and a Zuptor H710/3 horizontal mixer. The basal feed was formulated in accordance with nutritional recommendations for Ross 308 broiler chickens (Aviagen, Broiler Nutrition Specifications). The chemical composition and ingredient composition of the feed are presented in Table 1. The nutrient content of the diets was calculated based on the chemical composition of the raw feedstuffs and the metabolizable energy value.

**TABLE 1:** Ingredients and chemical composition of diets (as-fed basis) (%).

Percentage %			
Ingredient	Starter feed (days 0–10)	Grower feed (days 10–24)	Finisher feed (days 24–42)
Wheat	35.02	40.00	50.00
Soybean meal	34.02	29.40	22.88
Maize	22.54	17.21	10.03
Rapeseed oil	2.01	2.50	4.11
Lard	2.00	2.97	3.50
Rapeseed meal	1.00	5.00	7.00
Premix without coccidiostat^a^	1.00	1.00	1.00
Monocalcium phosphate	0.72	0.55	0.41
Calcium carbonate	0.62	0.44	0.26
L-Methionine	0.30	0.24	0.18
L-Lysine	0.22	0.18	0.20
Sodium chloride	0.20	0.20	0.20
NaHCO_3_	0.12	0.16	0.12
Threonine	0.12	0.08	0.07
L-Valine	0.12	0.06	0.03
OptiPhos® (0.01%)^b^	0.01	0.01	0.01
Calculated nutritional value			
AME_N_ MJ/kg	12.47	12.81	13.20
Crude protein	22.7	21.54	19.50
Crude fat	6.02	6.50	7.13
P available	0.48	0.42	0.36
Ca	0.96	0.75	0.65
Na	0.16	0.18	0.18
Cl	0.16	0.17	0.17
Lys dig	1.25	1.18	1.08
Met dig	0.6	0.51	0.48
Thr dig	0.84	0.79	0.72
Val dig	0.94	0.91	0.84

^a^ Vitamin–mineral premix provided per kg diet: Mn, 55 mg; Zn, 50 mg; Fe, 80 mg; Cu, 5 mg; Se, 0.1 mg; I, 0.36 mg; Na, 1.6 g, retinol, 2.48 mg; cholecalciferol, 25 μg; dl-ɑ-tocopherol, 60 mg; cyanocobalamin, 0.012 mg; menadione sodium bisulfite, 1.1 mg; niacin, 53 mg; choline chloride, 1,020 mg; folic acid, 0.75 mg; biotin, 0.25 mg; riboflavin, 5.5 mg; xylanase (Econase HCP, 4000; AB, vista, Marlborough, UK), 4 mg.

^b^ OptiPhos®: 6-phytase derived from *Escherichia coli*

### 2.5 Blood and tissue sample collection

Blood and tissues were sampled from three birds per replicate (30 samples in total) in each group. The samples were not pooled. Blood samples were collected on DOH from chicks killed by cervical dislocation (Murphy et al., 2016) (before feeding) and again from the wing vein at 7 days post-hatch. Blood was collected into tubes with a clotting activator (Vacuette, Medlab Products, Raszyn, Poland). The samples were thence transported to the laboratory at +4 °C within 1 h and centrifuged at room temperature (22 °C) for 15 min at 1,000 × g. The serum was stored at −80 °C until analysis.

Tissues were collected for cytokine, immunoglobulin and acute-phase protein analysis from the same chickens on the same days as the blood. On DOH, samples were collected from the liver (caudal part of the right liver lobe), small intestine (ileum–midsection between Meckel’s diverticulum and the ileocecal junction), large intestine (midsection between the ileo–cecal–colic junction and cloaca), and yolk sac (the contents of the yolk sac were collected after its removal according to Sloan (1936)). At 7 days post-hatch, tissues were collected from the liver and the small and large intestine. The tissues were washed in ice-cold saline (87 mM NaCl, 2.5 mM KCl, 1.25 mM NaH_2_PO_4_, 25 mM NaHCO_3_, 0.5 mM CaCl_2_, 7 mM MgSO_4_, 25 mM glucose, and 75 mM sucrose, pH 7.4) and stored at −80 °C for subsequent analysis. Tissue samples were not pooled.

At the same time, samples of the ileum (middle part of the ileum, 2 cm) were collected from the same birds and snap-frozen in liquid nitrogen. They were stored at −80 °C for immunoglobulin mRNA analysis.

### 2.6 Growth performance

Growth performance parameters, including body weight gain (BWG), feed intake (FI), and feed conversion ratio (FCR), were recorded on days 10, 24, and 42. Calculations were made for the starter (0–10 days), grower (10–24 days), and finisher (24–42 days) period, as well as for the cumulative experimental period (0–42 days). FI and FCR were recorded on a per-pen basis on days 10, 24, and 42. FCR for all experimental groups during the 42-day period was calculated as mean feed intake/mean weight. The body weight of the chicks after hatching and the mortality rate throughout the experiment were also determined.

### 2.7 Assays of acute-phase proteins and immunoglobulins in chicken serum and tissues

ELISA kits for chicken haptoglobin (Hp; catalog number E0016Ch), alpha 1-acid glycoprotein (α-1-AGP; catalog number EA0033Ch), and ceruloplasmin (catalog number EA0031Ch) were obtained from BT Lab (Bioassay Technology Laboratory), Shanghai, China. ELISA kits for chicken serum amyloid A (SAA) (catalog number orb561505) and mannose-binding lectin (MBL) (catalog number orb549452) were obtained from Biorbyt Ltd, Cambridge, United Kingdom.

ELISA kits for chicken IgM (catalog number E-EL-Ch0367), IgA (catalog number E-EL-Ch0620), and IgY (catalog number E-EL-Ch0001) were obtained from Elabscience Biotechnology Co., Ltd, Houston, USA. The assays were performed according to the manufacturer’s specifications. Levels of acute-phase proteins and immunoglobulins were determined on the day of hatch (DOH) in the serum, liver, small and large intestine, and yolk sac and on day 7 in the serum, liver, and small and large intestine. The α-1-AGP content was expressed as µg/mL total protein. Hp, ceruloplasmin, SAA, MBL, IgA, and IgY contents were expressed as ng/mL total protein. IgM content was expressed as pg/mL total protein.

### 2.8 Assays of cytokines in chicken serum and tissues

ELISA kits were used to determine IL-2, IL-12, TNF-α, TGF-β1 (Qayee-Bio, Shanghai, China, no. QY-E80014, QY-E80157, QY-E80030, and QY-E80041), IL-6, IL-8, IL-10, IFN-γ (EIAab Science Inc., Wuhan, Hubei, China, no. E0079c, E0080c, E0056c, and E0049c), and IL-1β (Wuhan Fine Biotech Co., Ltd., Wuhan, Hubei, China, no. ECH0040) in the chicken serum and tissues. All assays were performed according to the manufacturer’s instructions. All samples were tested in triplicate.

### 2.9 Measurement of organ weights

Immediately after blood sampling, the spleen, bursa of Fabricius, and thymus were excised, stripped of adhering tissue, and weighed individually. The weight was expressed as relative weight to live body weight. The entire procedure was performed as described by Kim et al. (2016).

### 2.10 mRNA expression of immunoglobulins

Total RNA was prepared from the homogenized snap-frozen ileum samples of each bird using the RNeasy mini kit (QIAGEN, Crawley, United Kingdom) following the manufacturer’s instructions. Quality and purity were assessed using the ND-1000 spectrophotometer at 260 nm/280 nm (NanoDrop Technologies, Silverside, Wilmington, DE, USA). Reverse transcription-PCR (qRT-PCR) was performed using the SuperScript II first-strand cDNA synthesis kit according to the manufacturer’s instructions (Invitrogen, Chorzów, Poland). All procedures are described in Jarosz et al. (2017b).

The expression of immunoglobulins IgA, IgY, and IgM was determined by qRT-PCR using the Applied Biosystems 7500 real-time PCR system (Applied Biosystems, Warrington, United Kingdom) (Lammers et al., 2010).

The primer sequences used for qRT-PCR are listed in Table 2. Each sample was subjected to qRT-PCR in triplicate, and the mean values were used for analysis. The threshold cycle (CT) values for the genes of interest were normalized to the average CT value of the housekeeping genes, and the relative expression of each replicate was calculated as 2^−ΔΔCT^ (Applied Biosystems user Bulletin #2; AI prism 7700 detection system, 2001). The reference gene used was β-actin (housekeeping gene). CT values for each sample were standardized for β-actin RNA. All research procedures have been described in detail by Pfaffl (2001) and Moreira Filho et al. (2018).

**TABLE 2:** Primer sequences.

Gene	Primer sequence (5′→ 3′)	Accession number
IgY	F: ATC-ACG-TCA-AGG-GAT-GCC-CG R: ACC-AGG-CAC-CTC-AGT-TTG-G	X07174.1
IgA	F: GTC-ACC-GTC-ACC-TGG-ACT-ACA R: ACC-GAT-GGT-CTC-CTT-CAC-ATC	S40610
IgM	F: GCA-TCA-GCG-TCA-CCG-AAA-GC R: TCC-GCA-CTC-CAT-CCT-CTT-GC	X01613.1
β-Actin	F: ACCACTGGCATTGTCATGGACTCT R: TCCTTGATGTCACGGACGATTTCC	NM_205518

F, forward; R, reverse.

### 2.11 Statistical analysis

Statistical analysis of the results was performed using the Statistica 13.2 PL package (StatSoft, Krakow, Poland). The significance of differences between the control (I) and experimental groups (II–IV) was tested for the levels of selected cytokines, immunoglobulins, acute-phase proteins, relative weight of lymphoid organs, and mRNA expression ratio in the ileum for the three immunoglobulins IgA, IgY, and IgM at two time points in early chicken life: DOH and 7 days post hatching (dependent variables). After evaluating data distribution and the equality of variances, one-way analysis of variance (ANOVA) with Tukey’s *post hoc* test was used for comparisons between experimental groups at a given age, and the t-test was deployed to analyze differences within the same group between the two analytical points. The same letters in tables indicate the absence of statistically significant differences. Results are expressed as mean and ±standard deviation (±SD), and differences were considered significant at p ≤ 0.05. The results are presented in Tables 3–8.

**TABLE 3:** Comparison of levels of selected cytokines determined in chickens from control (I) and experimental groups (II–IV) using one-way ANOVA. Results are presented as means and standard deviation (±SD) at p ≤ 0.05. DOH, on the day of hatch.

Cytokine	Group	DOH					7 days			
		Serum	Liver	Small intestine	Large intestine	Yolk sac	Serum	Liver	Small intestine	Large intestine
IL-1β (pg/mL)	I	50.03 ± 7.96^ab^	2687.51 ± 1924.09^ab^	57.19 ± 26.82^a^	6.29 ± 3.01^a^	289.48 ± 62.55^a^	111.86 ± 44.61^a^	5083.15 ± 311.79^ab^	389.37 ± 36.11^a^	289.6 ± 36.97^a^
	II	21.63 ± 10.11^c^	5222.63 ± 94.24^a^	55.94 ± 23.23^a^	21.64 ± 7.41^a^	467.78 ± 161.92^b^	17.82 ± 2.83^b^	5243.29 ± 97.77^a^	354.63 ± 65.29^a^	518.2 ± 74.51^b^
	III	40.92 ± 0.71^ab^	4989.65 ± 32.24^ab^	163.25 ± 18.63^b^	19.09 ± 1.32^a^	222.7 ± 38.16^a^	50.93 ± 4.83^b^	5179.78 ± 121.79^ab^	345.36 ± 15.71^a^	548.8 ± 54.69^b^
	IV	58.82 ± 12.7^a^	474.86 ± 139.69^b^	46.58 ± 16.68^a^	212.2 ± 39.97^b^	209.53 ± 69.21^a^	30.32 ± 6.63^b^	4838.25 ± 204.95^b^	255.68 ± 50.69^b^	477.41 ± 46.98^b^
p-value		<0.0001	<0.0001	0.002	<0.0001	<0.0001	<0.0001	0.01	0.004	0.003
IL-2 (ng/mL)	I	12.2 ± 5.52^ab^	127.43 ± 11.95^a^	15.67 ± 1.73^a^	12.26 ± 2.67^a^	12.74 ± 3.27^a^	7.36 ± 1.55^ab^	80.33 ± 3.9^a^	15.79 ± 3.06^a^	9.26 ± 2.83^a^
	II	10.81 ± 5.16^ab^	87.29 ± 8.18^b^	15.67 ± 2.79^a^	14.35 ± 1.5^a^	12.45 ± 2.71^a^	3.62 ± 1.05^a^	96.59 ± 3.09^a^	21.42 ± 3.53^b^	13.12 ± 0.64^a^
	III	4.88 ± 1.28^a^	82.9 ± 5.06^b^	27.73 ± 1.65^b^	10.59 ± 3.36^a^	7.64 ± 1.25^b^	3.78 ± 0.61^a^	80.88 ± 19.52^a^	16.06 ± 2.26^a^	20.52 ± 2.84^b^
	IV	16.32 ± 4.95^b^	59.78 ± 4.43^c^	25.39 ± 3.63^b^	39.06 ± 11.52^b^	16.53 ± 3.14^a^	11.91 ± 5.51^b^	94.26 ± 7.36^a^	18.57 ± 2.59^ab^	17.93 ± 2.84^b^
p-value		0.007	<0.0001	<0.0001	<0.0001	0.005	0.006	0.4	0.02	<0.0001
IL-6 (pg/mL)	I	3242.72 ± 351.61^a^	3208.46 ± 131.08^a^	82.92 ± 9.49^a^	109.72 ± 32.29^ab^	191.17 ± 51.99^a^	1445.19 ± 148.66^a^	3017.96 ± 67.14^ab^	287.42 ± 99.84^ab^	381.31 ± 48.05^a^
	II	2350.71 ± 619.27^b^	3511.36 ± 207.98^b^	88.04 ± 41.9^a^	84.53 ± 29.96^ab^	1359.08 ± 509.22^b^	609.33 ± 68.94^a^	3250.99 ± 20.21^a^	202.07 ± 37.59^ab^	495.09 ± 138.79^ac^
	III	2086.04 ± 311.78^b^	1137.33 ± 78.39^c^	137.22 ± 20.5^b^	49.36 ± 17.6^a^	907.82 ± 142.4^b^	1782.39 ± 115.68^a^	2750.83 ± 361.18^b^	179.94 ± 16.28^a^	717.53 ± 226.55^b^
	IV	3614.73 ± 141.05^a^	3038.35 ± 85.13^a^	28.3 ± 11.51^c^	132.75 ± 49.16^b^	1168.01 ± 77.46^b^	2185.35 ± 55.85^a^	2930.5 ± 98.68^ab^	311.87 ± 94.4^b^	391.19 ± 68.08^c^
p-value		<0.0001	<0.0001	<0.0001	0.007	<0.0001	0.5	0.004	0.02	0.003
IL-8 (pg/mL)	I	1718.19 ± 393.91^a^	769.49 ± 211.75^a^	132.34 ± 8.54^a^	88.67 ± 5.21^a^	80.33 ± 16.98^a^	358.21 ± 110.42^a^	1588.46 ± 47.26^a^	182.81 ± 78.53^ab^	145.81 ± 11.41^a^
	II	883.18 ± 518.05^b^	1842.43 ± 63.01^b^	85.21 ± 12.78^b^	64.74 ± 6.25^a^	118.31 ± 61.29^a^	156.92 ± 90.15^b^	1466.75 ± 204.39^a^	219.34 ± 42.69^a^	209.39 ± 33.31^c^
	III	229.44 ± 119.08^b^	613.47 ± 78.47^a^	115.28 ± 7.68^a^	71.68 ± 19.79^a^	122.4 ± 66.74^a^	54.93 ± 18.1^b^	2550.22 ± 288.76^b^	129.79 ± 27.65^b^	256.97 ± 25.7^bc^
	IV	2105 ± 472.02^a^	1348.01 ± 95.62^c^	151.93 ± 16.45^a^	82.29 ± 26.21^a^	257.79 ± 5.08^b^	684.77 ± 55.87^c^	1403.33 ± 62.81^a^	195.29 ± 19.32^ab^	286.45 ± 42.94^b^
p-value		<0.0001	<0.0001	<0.0001	0.14	0.003	0.003	0.008	0.02	<0.0001
IL-10 (pg/mL)	I	1521.42 ± 546.46^a^	7407.56 ± 176.91^a^	110.7 ± 54.99^a^	67.84 ± 17.08^a^	832.22 ± 357.25^a^	145.34 ± 85.59^a^	7046.02 ± 667.56^ab^	853.59 ± 444.08^ab^	1242.18 ± 356.25^a^
	II	1386.39 ± 680.52^ab^	7483.41 ± 289.48^a^	217.88 ± 70.55^ab^	96.96 ± 21.9^ab^	975.73 ± 534.26^a^	75.2 ± 7.79^a^	7435.1 ± 320.29^a^	1083.95 ± 525.65^ab^	1752.28 ± 57.94^ab^
	III	516.19 ± 208.23^b^	4586.4 ± 134.03^b^	424.24 ± 157.14^b^	129.41 ± 29.9^b^	463.41 ± 36.41^a^	121.26 ± 97.74^a^	6650.28 ± 255.04^b^	627.9 ± 54.88^a^	2468.45 ± 531.25^b^
	IV	1786.3 ± 571.19^a^	3504.83 ± 2327.4^b^	55.45 ± 24.65^a^	402.92 ± 49.79^c^	1197.24 ± 71.54^a^	215.9 ± 123.76^a^	7115.58 ± 185.92^ab^	1605.8 ± 392.85^b^	1266.58 ± 129.41^a^
p-value		0.001	<0.0001	<0.0001	<0.0001	0.1	0.7	0.02	0.03	0.007
IL-12 (ng/mL)	I	94.09 ± 4.73^a^	274.74 ± 40.19^a^	47.12 ± 3.3^a^	56.93 ± 6.55^a^	73.62 ± 6.21^ac^	82.31 ± 3.89^a^	132.85 ± 9.64^a^	67.04 ± 5.29^a^	48.15 ± 6.33^a^
	II	82.47 ± 3.9^b^	168.93 ± 22.37^b^	56.54 ± 3.76^b^	49.26 ± 1.56^b^	82.84 ± 7.99^c^	72.63 ± 7.43^a^	190.88 ± 4.78^b^	65.21 ± 1.74^a^	71.27 ± 0.76^b^
	III	83.93 ± 1.51^ab^	256.85 ± 28.48^a^	61.74 ± 2.1^b^	49.72 ± 3.35^b^	62.51 ± 1.11^b^	90.99 ± 3.27^ab^	189.13 ± 24.03^b^	82.5 ± 1.34^b^	83.46 ± 3.15^c^
	IV	90.93 ± 12.02^ab^	138.62 ± 16.99^b^	70.8 ± 7.25^c^	73.21 ± 2.66^c^	72.23 ± 2.51^a^	95.66 ± 10.11^b^	180.46 ± 19.34^b^	80.72 ± 7.37^b^	88.05 ± 3.43^c^
p-value		0.03	<0.0001	<0.0001	0.006	0.02	<0.0001	0.007	<0.0001	<0.0001
TNF-α (ng/mL)	I	56.73 ± 4.9^a^	550.2 ± 106.76^a^	132.87 ± 26.7^a^	66.35 ± 8.56^a^	52.41 ± 10.06^a^	20.27 ± 2.09^a^	204.23 ± 46.52^a^	81.56 ± 30.69^a^	117.72 ± 13.49^ab^
	II	30.76 ± 4.89^ab^	356.34 ± 23.12^ab^	61.54 ± 11.5^b^	123.5 ± 16.06^a^	60.14 ± 7.14^ab^	6.76 ± 0.89^b^	253.08 ± 44.32^abc^	102.13 ± 4.3^a^	101.48 ± 43.84^a^
	III	19.69 ± 3.43^b^	319.96 ± 29.16^b^	72.67 ± 9.29^bc^	85.08 ± 8.1^a^	41.7 ± 14.9^b^	16.32 ± 1.73^ab^	343.97 ± 22.48^ac^	106.55 ± 20.18^a^	181.5 ± 16.24^b^
	IV	75.13 ± 10.89^ac^	259.55 ± 79.18^b^	128.65 ± 24.72^ac^	195.68 ± 19.1^a^	93.84 ± 39.19^a^	72.37 ± 10.56^c^	371.58 ± 69.12^b^	98.33 ± 36.45^a^	110.35 ± 45.11^ab^
p-value		0.004	<0.0001	<0.0001	0.7	<0.0001	<0.0001	<0.0001	0.8	0.002
IFN-γ (pg/mL)	I	2122.28 ± 620.21^ab^	2551.24 ± 709.07^a^	144.67 ± 8.49^a^	103.84 ± 38.32^ab^	64.83 ± 15.23^a^	25.26 ± 6.08^ab^	2429.4 ± 280.1^a^	467.7 ± 111.08^a^	509.9 ± 65.11^a^
	II	1293.09 ± 842.93^ab^	4183.56 ± 232.94^b^	88.73 ± 13.73^ab^	37.84 ± 10.17^a^	226.25 ± 55.48^b^	11.82 ± 1.57^a^	2841.97 ± 58.9^ab^	424.03 ± 7.75^a^	381.61 ± 72.66^a^
	III	367.73 ± 191.37^b^	1470.88 ± 207.9^c^	41.11 ± 4.2^b^	96.11 ± 29.73^ab^	89.28 ± 17.89^ab^	92.14 ± 64.37^bc^	2280.03 ± 213.1^c^	376.04 ± 23.69^ab^	519.96 ± 163.62^a^
	IV	2591.68 ± 775.47^a^	2728.03 ± 510.74^a^	141.4 ± 19.49^a^	195.15 ± 37.25^b^	285.59 ± 77.69^b^	304.65 ± 43.61^bc^	3305.02 ± 111.75^ab^	282.48 ± 74.27^b^	835.91 ± 84^b^
p-value		0.002	<0.0001	<0.0001	<0.0001	<0.0001	<0.0001	<0.0001	0.003	0.004
TGF-β1 (pg/mL)	I	9.63 ± 1.50^a^	45.73 ± 3.07^a^	6.5 ± 0.81^a^	6.77 ± 0.56^a^	8.15 ± 0.33^a^	8.60 ± 0.74^a^	29.39 ± 0.62^a^	10.55 ± 0.65^a^	9.15 ± 2.08^a^
	II	9.05 ± 1.13^a^	31.12 ± 1.61^b^	8.18 ± 0.66^b^	6.84 ± 0.38^a^	10.96 ± 2.03^b^	7.57 ± 0.76^a^	37.53 ± 0.4^b^	10.99 ± 0.4^a^	11.51 ± 0.63^b^
	III	9.14 ± 0.43^a^	43.14 ± 1.82^a^	8.75 ± 0.18^b^	7.83 ± 0.24^b^	8.59 ± 1.37^a^	7.85 ± 0.53^ab^	31.26 ± 2.16^a^	10.29 ± 0.59^a^	13.45 ± 0.63^bc^
	IV	9.09 ± 0.79^a^	27.66 ± 2.56^b^	9.27 ± 0.73^b^	12.61 ± 0.29^c^	10.05 ± 0.36^ab^	10.11 ± 1.09^b^	30.34 ± 2.32^a^	11.41 ± 2.17^a^	10.64 ± 0.11^ab^
p-value		0.08	<0.0001	0.003	<0.0001	0.02	0.001	0.004	0.7	0.005

a, b, c – statistical differences.

**TABLE 4:** Comparison of levels of selected acute-phase proteins determined in chickens from control (I) and experimental groups (II–IV) using one-way ANOVA. Results are presented as means and standard deviation (±SD) at p ≤ 0.05. DOH, on the day of hatch.

Protein	Group	DOH					7 days			
		Serum	Liver	Small intestine	Large intestine	Yolk sac	Serum	Liver	Small intestine	Large intestine
α-1-AGP (µg/mL)	I	206.92 ± 25.30^a^	4443.39 ± 149.62^a^	13,055.65 ± 868.14^a^	13,350.77 ± 356.74^a^	3196.03 ± 669.65^a^	404.92 ± 54.69^a^	3246.75 ± 78.67^ac^	1720.46 ± 79.65^ac^	2310.75 ± 248.44^ab^
	II	307.83 ± 63.54^ab^	2458.96 ± 190.01^b^	8686.51 ± 3022.75^ab^	10,645.33 ± 3165.23^ab^	4116.11 ± 676.09^ab^	610.70 ± 36.79^ab^	3901.98 ± 44.66^b^	1560.91 ± 90.31^a^	2613.26 ± 151.10^a^
	III	312.36 ± 127.36^ab^	4280.73 ± 72.77^a^	5158.68 ± 146.31^b^	13,732.40 ± 172.87^a^	4924.85 ± 606.63^b^	750.03 ± 49.48^b^	3552.5 ± 165.76^ab^	1011.2 ± 169.75^b^	2504.91 ± 496.84^a^
	IV	615.99 ± 343.06^b^	3763.94 ± 487.43^bc^	12,255.15 ± 970.89^a^	4481.64 ± 222.95^b^	4904.53 ± 615.75^b^	382.1 ± 136.81^a^	2209.11 ± 136.56^c^	2356.34 ± 121.51^c^	1552.85 ± 242.09^b^
p-value		0.02	<0.0001	0.001	0.002	0.003	<0.0001	<0.0001	<0.0001	0.0015
Haptoglobin (ng/mL)	I	78.02 ± 14.46^a^	85.44 ± 1.39^a^	15.69 ± 2.58^a^	37.25 ± 6.95^ac^	77.55 ± 6.78^a^	133.72 ± 4.78^a^	99.81 ± 12.68^ab^	78.20 ± 2.12^ab^	56.74 ± 12.79^ab^
	II	85.27 ± 10.21^a^	106.67 ± 23.22^ab^	65.22 ± 14.82^bc^	46.18 ± 2.13^a^	68.93 ± 7.63^a^	133.83 ± 11.05^a^	96.23 ± 8.24^ab^	73.38 ± 7.46^a^	46.39 ± 6.59^a^
	III	84.97 ± 35.23^a^	111.16 ± 4.90^b^	23.34 ± 4.09^a^	16.34 ± 5.70^c^	72.20 ± 6.69^a^	120.03 ± 13.02^a^	86.13 ± 2.99^a^	96.02 ± 6.23^b^	80.27 ± 2.15^b^
	IV	88.49 ± 25.96^a^	102.21 ± 0.87^ab^	32.77 ± 1.87^ac^	56.17 ± 3.50^b^	71.79 ± 6.96^a^	130.02 ± 4.15^a^	122.14 ± 1.24^b^	63.03 ± 5.85^a^	58.20 ± 2.41^ab^
p-value		0.7	0.0015	<0.0001	<0.0001	0.3	0.15	0.001	<0.0001	0.001
Ceruloplasmin (ng/mL)	I	354.63 ± 133.94^a^	1284.45 ± 76.66^a^	2789.06 ± 331.78^a^	2674.71 ± 90.46^a^	580.61 ± 83.89^a^	1106.06 ± 145.99^a^	988.15 ± 84.52^a^	526.25 ± 39.16^a^	513.88 ± 53.20^a^
	II	455.44 ± 188.29^b^	586.49 ± 10.17^b^	1954.38 ± 796.85^b^	2343.74 ± 505.89^ab^	830.51 ± 200.85^ab^	513.18 ± 261.1^ab^	932.31 ± 102.81^ab^	615.84 ± 167.91^a^	722.19 ± 15.20^b^
	III	213.81 ± 21.91^c^	1162.39 ± 217.08^a^	1979.6 ± 103.18^b^	3084.41 ± 68.46^a^	1469.75 ± 409.79^b^	108.92 ± 58.65^b^	953.58 ± 122.37^a^	487.10 ± 78.90^a^	758.79 ± 116.73^b^
	IV	175.45 ± 60^c^	1113.24 ± 141.88^ab^	2439.14 ± 105.58^a^	1043.5 ± 287.10^b^	1180.06 ± 36.27^b^	1221.14 ± 152.24^a^	527.24 ± 46.09^b^	665.54 ± 111.66^a^	531.02 ± 61.01^a^
p-value		0.0014	0.02	0.013	<0.0001	<0.0001	<0.0001	0.004	0.6	0.001
SAA (ng/mL)	I	2.74 ± 0.18^ab^	11.19 ± 0.22^a^	2.51 ± 0.20^a^	4.77 ± 0.52^a^	6.08 ± 0.51^a^	7.55 ± 0.24^a^	11.13 ± 0.25^a^	6.92 ± 0.55^a^	4.75 ± 0.14^a^
	II	3.87 ± 0.78^ab^	10.85 ± 0.89^a^	4.63 ± 0.21^b^	4.42 ± 0.81^a^	4.78 ± 1.34^b^	7.25 ± 0.46^a^	11.17 ± 0.16^a^	6.99 ± 0.32^a^	5.48 ± 0.26^b^
	III	4.40 ± 1.93^a^	10.96 ± 0.04^a^	3.15 ± 0.34^b^	1.86 ± 0.99^b^	8.61 ± 1.14^c^	5.03 ± 0.82^b^	7.72 ± 2.17^b^	6.99 ± 0.36^a^	4.77 ± 0.47^a^
	IV	2.13 ± 0.29^b^	11.64 ± 0.39^a^	4.24 ± 0.5^c^	3.78 ± 1.02^a^	4.06 ± 0.48^b^	7.17 ± 0.53^a^	11.27 ± 0.19^a^	7.81 ± 0.15^b^	8.19 ± 0.55^c^
p-value		0.002	0.5	<0.0001	<0.0001	<0.0001	0.003	0.008	0.01	0.006
MBL (ng/mL)	I	23.09 ± 4.96^ab^	59.06 ± 4.46^a^	2.95 ± 0.56^a^	2.31 ± 0.35^a^	4.60 ± 0.77^a^	13.00 ± 2.48^a^	130.59 ± 30.64^ab^	5.34 ± 2.24^a^	6.91 ± 30^a^
	II	16.59 ± 3.56^a^	214.17 ± 6.30^b^	2.96 ± 0.43^a^	2.77 ± 0.54^ab^	26.54 ± 12.40^b^	11.01 ± 4.72^ab^	147.52 ± 3.07^ab^	4.83 ± 0.23^a^	7.45 ± 0.26^ab^
	III	18.03 ± 7.98^ab^	76.97 ± 25.81^a^	3.45 ± 0.26^ab^	3.29 ± 0.25^ab^	11.57 ± 0.71^b^	9.96 ± 0.77^b^	88.81 ± 38.57^a^	6.75 ± 1.44^a^	12.38 ± 1.29^ab^
	IV	25.09 ± 2.13^b^	189.45 ± 14.29^ab^	3.68 ± 0.33^b^	3.53 ± 0.22^b^	9.69 ± 0.96^ab^	26.84 ± 0.57^c^	152.36 ± 13.42^b^	4.64 ± 1.01^a^	13.04 ± 1.15^b^
p-value		0.02	<0.0001	0.01	0.002	<0.0001	0.002	0.02	0.3	<0.0001

a, b, c – statistical differences.

**TABLE 5:** Comparison of levels of selected immunoglobulins determined in chickens from control (I) and experimental groups (II–IV) using one-way ANOVA. Results are presented as means and standard deviation (±SD) at p ≤ 0.05. DOH, on the day of hatch.

Immunoglobulin	Group	DOH					7 days			
		Serum	Liver	Small intestine	Large intestine	Yolk sac	Serum	Liver	Small intestine	Large intestine
IgA (ng/mL)	I	0.39 ± 0.13^a^	54.24 ± 3.45^a^	3.99 ± 1.40^a^	2.08 ± 0.13^a^	0.28 ± 0.01^a^	1.14 ± 0.23^a^	36.02 ± 5.45^a^	5.14 ± 0.56^a^	2.59 ± 0.41^a^
	II	0.57 ± 0.21^a^	76.32 ± 2.65^b^	1.91 ± 0.06^b^	5.56 ± 0.74^b^	0.42 ± 0.01^b^	2.05 ± 0.74^ab^	51.34 ± 3.41^b^	5.68 ± 0.42^a^	4.58 ± 0.35^b^
	III	0.47 ± 0.04^a^	60.24 ± 2.32^c^	3.80 ± 0.21^a^	2.22 ± 0.36^a^	0.48 ± 0.13^b^	1.91 ± 1.07^ab^	34.57 ± 2.45^a^	5.70 ± 1.43^a^	4.15 ± 0.36^b^
	IV	1.02 ± 0.33^b^	77.74 ± 2.38^b^	4.25 ± 0.50^a^	3.16 ± 0.38^c^	0.24 ± 0.09^a^	2.85 ± 0.37^b^	41.11 ± 3.15^a^	4.36 ± 0.19^b^	6.74 ± 0.25^c^
p-value		<0.0001	<0.0001	<0.0001	<0.0001	<0.0001	0.008	<0.0001	<0.0001	0.005
IgM (pg/mL)	I	3.18 ± 0.12^a^	35.30 ± 0.55^a^	2.18 ± 0.71^a^	3.21 ± 0.08^a^	9.55 ± 0.29^a^	10.34 ± 0.64^a^	33.77 ± 0.34^a^	14.68 ± 1.0^a^	10.13 ± 0.50^a^
	II	8.86 ± 0.23^b^	35.5 ± 0.38^a^	5.95 ± 2.28^b^	3.19 ± 0.81^a^	8.37 ± 1.15^ab^	12.86 ± 0.50^a^	25.04 ± 5.69^b^	11.44 ± 0.77^bc^	16.54 ± 3.83^b^
	III	4.85 ± 1.62^c^	35.64 ± 0.29^a^	5.35 ± 0.24^b^	1.87 ± 0.62^b^	8.73 ± 0.25^ab^	4.55 ± 0.77^b^	35.44 ± 0.19^a^	11.93 ± 0.77^b^	10.72 ± 0.79^a^
	IV	2.59 ± 0.32^a^	35.51 ± 0.51^a^	6.68 ± 0.81^b^	6.63 ± 0.87^c^	7.58 ± 1.61^b^	12.54 ± 3.03^a^	31.79 ± 0.57^a^	9.65 ± 1.62^c^	14.72 ± 2.68^a^
p-value		<0.0001	0.7	<0.0001	<0.0001	0.04	<0.0001	<0.0001	<0.0001	<0.0001
IgY (pg/mL)	I	7.64 ± 0.21^a^	89.29 ± 5.50^a^	4.50 ± 0.94^a^	22.18 ± 4.60^a^	262.31 ± 177.90^a^	13.76 ± 2.09^a^	54.20 ± 5.29^a^	5.79 ± 2.66^a^	6.55 ± 1.84^a^
	II	10.86 ± 4.11^a^	101.35 ± 1.43^a^	6.60 ± 1.70^a^	23.68 ± 1.70^a^	41.28 ± 4.28^b^	4.94 ± 2.33^b^	71.77 ± 3.30^b^	2.81 ± 0.41^a^	17.40 ± 5.18^a^
	III	25.78 ± 9.82^b^	172.72 ± 12.79^b^	16.32 ± 3.33^b^	11.21 ± 0.60^b^	259.67 ± 121.87^a^	3.48 ± 0.49^b^	80.38 ± 3.68^c^	5.95 ± 1.05^a^	9.26 ± 1.25^b^
	IV	1.78 ± 0.35^c^	120.52 ± 16.38^c^	14.72 ± 2.49^b^	18.26 ± 0.38^a^	94.38 ± 9.89^ab^	2.40 ± 0.92^b^	113.16 ± 3.54^d^	43.3 ± 6.38^b^	41.14 ± 6.14^a^
p-value		<0.0001	<0.0001	<0.0001	<0.0001	<0.0001	<0.0001	<0.0001	<0.0001	<0.0001

a, b, c – statistical differences.

**TABLE 6:** Comparison of mean mRNA expression ratio values for selected immunoglobulins determined in the ileum of chickens from control (I) and experimental groups (II–IV) using one-way ANOVA. Results are presented as means and standard deviation (±SD) at p ≤ 0.05. DOH, on the day of hatch.

Group	Ileum mRNA expression ratio					
	DOH			7 days		
	IgA	IgM	IgY	IgA	IgM	IgY
I	1.15 ± 0.10^a^	1.20 ± 0.21^a^	1.35 ± 0.19^a^	1.54 ± 0.24^a^	2.20 ± 0.27^a^	2.30 ± 0.27^b^
II	1.15 ± 0.13^a^	1.10 ± 0.10^a^	1.15 ± 0.13^a^	2.42 ± 0.42^b^	2.58 ± 0.46^ab^	2.72 ± 1.06^b^
III	1.12 ± 0.13^a^	1.28 ± 0.24^a^	1.21 ± 0.21^a^	3.20 ± 0.47^c^	2.26 ± 0.31^ab^	3.14 ± 0.78^b^
IV	1.10 ± 0.12^a^	1.25 ± 0.10^a^	1.20 ± 0.00^a^	2.28 ± 0.48^b^	2.38 ± 0.49^b^	3.40 ± 0.49^b^
p-value	0.95	0.19	0.2	0.00003	0.039	0.27

a, b, c – statistical differences.

**TABLE 7:** Chicken growth performance. Results are presented as means and standard deviation (±SD) at p ≤ 0.05.

	Initial body weight	Starter (0–10)			Grower (10–24)			Finisher (24–42)			Total (0–42)		
Group		BWG	FI	FCR	BWG	FI	FCR	BWG	FI	FCR	BWG	FI	FCR
I	41.28 ± 2.32^a^	413 ± 18.8^ab^	487 ± 25.3^a^	1.18 ± 0.04^a^	1136 ± 27.8^a^	1551 ± 9.1^a^	1.366 ± 0.03^a^	1342 ± 126^a^	2591 ± 147^a^	1.93 ± 0.12	2890 ± 131^a^	4629 ± 154^a^	1.60 ± 0.06
II	41.57 ± 2.13^a^	401 ± 15.9^b^	459 ± 15.0^b^	1.14 ± 0.5^b^	1052 ± 25.6^b^	1463 ± 28.6^c^	1.39 ± 0.02^b^	1271 ± 112^b^	2349 ± 153^b^	1.85 ± 0.16	2724 ± 140^b^	4271 ± 155^b^	1.57 ± 0.07
III	41.79 ± 2.33^a^	424 ± 19.0^a^	471 ± 29.1^ab^	1.11 ± 0.05^c^	1120 ± 31.5^a^	1518 ± 23.2^b^	1.36 ± 0.02^a^	1338 ± 160^a^	2503 ± 342^ab^	1.88 ± 0.25	2881 ± 151^a^	4492 ± 325^a^	1.56 ± 0.12
IV	41.12 ± 2.17^a^	416 ± 18.9^ab^	484 ± 23.2^a^	1.17 ± 0.03^ab^	1121 ± 35.4^a^	1539 ± 48.3^ab^	1.373 ± 0.03^ab^	1354 ± 67.4^a^	2498 ± 150^ab^	1.85 ± 0.11	2891 ± 82^a^	4522 ± 181^a^	1.56 ± 0.05
P-value	0.7433	0.0465	0.013	0.0005	<0.0001	<0.0001	0.0423	0.0494	0.0277	0.3313	<0.0001	0.0005	0.3463

a, b, c, statistical differences; BWG, body weight gain; FI, feed intake; FCR, feed conversion ratio.

**TABLE 8:** Results of one-way analysis of variance (ANOVA) and Tukey’s test for relative weight of lymphoid organs (grams–g) in chickens^a^. Results are expressed as means and standard deviation (±SD) at p ≤ 0.05.

Organ (g)	Group									
	I	II	III	IV	p-value	I	II	III	IV	p-value
	DOH					7 days				
Bursa of Fabricius	0.09 ± 0.01^a^	0.1 ± 0.01^a^	0.09 ± 0.01^a^	0.09 ± 0.02^a^	0.8	0.17 ± 0.02^a^	0.18 ± 0.03^a^	0.19 ± 0.02^a^	0.18 ± 0.01^a^	0.7
Thymus	0.04 ± 0.01^a^	0.04 ± 0.01^a^	0.05 ± 0.02^a^	0.04 ± 0.01^a^	0.5	0.37 ± 0.06^a^	0.39 ± 0.08^a^	0.42 ± 0.04^a^	0.39 ± 0.05^a^	0.6
Spleen	0.02 ± 0.01^a^	0.03 ± 0.01^a^	0.03 ± 0.01^a^	0.03 ± 0.01^a^	0.1	0.05 ± 0.02^a^	0.09 ± 0.01^b^	0.07 ± 0.03^ab^	0.06 ± 0.01^ab^	0.02

^a^ Values (g/100 g of body weight) are means from three birds per replicate (30 samples in total) from each group. Lowercase letters (a, b) indicate statistically significant differences (p ≤ 0.05) between groups. DOH, on the day of hatch.

## 3 Results

### 3.1 Chicken growth performance

In the period from 0 to 10 days of life, the BWG was statistically significantly higher in group III than in groups II and I (control). In the periods from 10 to 24 and from 24 to 42 days of life, BWG was statistically significantly lower in group II than in the other groups. Similarly, BWG in group II was statistically significantly lower than in the other groups during the cumulative experimental period (days 0–42). During this period (days 0–42), FI was statistically significantly lower in group II than in the other groups. From 0 to 10 days of age, FI was statistically significantly lower in group II than in the groups I and IV. FI in group II was also statistically significantly lower than in the other groups from 10 to 24 days of age and statistically significantly lower than in control group (I) from 24 to 42 days of age. FCR in group III was statistically significantly lower than in the other groups from 0 to 10 days of age. In the period between 10 and 24 days of life, a statistically significantly higher FCR was recorded in group II compared to groups I and III (Table 7). No statistically significant differences in the initial body weight of chicks were observed between the groups (Table 7). Compared to the control group, a significantly higher total mortality rate of chicks was observed in group II (3.33%) (Supplementary Table S1).

### 3.2 Levels of acute-phase proteins in chicken serum and tissues

On DOH, the content of α-1-AGP in the serum of chickens in group IV was statistically significantly higher than in the control group. At 7 days post-hatch, the serum content of α-1-AGP was statistically significantly higher in group III than in groups I and IV. Analysis of α-1-AGP in chicken livers on DOH showed its statistically significantly higher content in the I and III groups than in groups II and IV. At 7 days, the content of α-1-AGP in the chicken liver was statistically significantly higher in group II than in groups I and IV. The content of α-1-AGP in the small intestine on DOH was statistically significantly lower in group III than in the other groups. Similarly, after 7 days, the content of α-1-AGP was statistically significantly lower in group III than in the other groups. On DOH, the content of α-1-AGP in the large intestine was statistically significantly lower in group IV than in groups I and III. After 7 days, the content of α-1-AGP in the large intestine was statistically significantly lower in group IV than in groups II and III. The content of α-1-AGP in the yolk sac was statistically significantly higher in groups III and IV than in the control group (I) (Table 4).

The Hp content in the livers of birds in group III on DOH was statistically significantly higher than in the control group. At 7 days, the Hp content in the liver of chickens was statistically significantly lower in group III than in group IV. On DOH, the content of Hp in the small intestine was statistically significantly higher in group II than in groups I and III. At 7 days, the content of Hp in the small intestine was statistically significantly higher in group III than in groups II and IV. The content of HP in the large intestine on DOH was statistically significantly higher in group IV than in the other groups. At 7 days, the content of Hp in the large intestines of birds in group III was statistically significantly higher than in group II (Table 4).

The ceruloplasmin content in the serum of chickens on DOH was statistically significantly lower in groups III and IV than in groups I and II. At 7 days, the serum ceruloplasmin content was statistically significantly lower in group III than in groups I and IV. In turn, its content in the livers of chickens on DOH was statistically significantly lower in group II than in groups I and III. At 7 days, the ceruloplasmin content in the liver was statistically significantly lower in group IV than in groups I and III. On DOH, statistically significantly lower ceruloplasmin contents in the small intestine were determined in groups II and III compared to groups I and IV. On DOH, ceruloplasmin content in the large intestine was statistically significantly lower in group IV than in groups I and III. At 7 days, it was statistically significantly lower in groups I and IV than in groups II and III. The ceruloplasmin content in the yolk sac was statistically significantly lower in group I than in groups III and IV (Table 4).

On DOH, the content of SAA in the serum of chickens was statistically significantly lower in group IV than in group IIII. At 7 days, the serum content of SAA was statistically significantly lower in group III than in the other groups. At 7 days, the SAA content in the liver was statistically significantly lower in group III than in the other groups. On DOH, the SAA content in the small intestine was statistically significantly lower in the control group (I) than in the other groups, whereas at 7 days it was statistically significantly higher in group IV than in the other groups. On DOH, the SAA content in the large intestine was statistically significantly lower in group III than in the other groups. At 7 days, it was statistically significantly higher in group IV than in the other groups. SAA content in the yolk sac was statistically significantly higher in group III than in the other groups (Table 4).

On DOH, the content of MBL in the serum of chickens in group IV was statistically significantly higher than in group II. After 7 days, the serum MBL content in group IV was statistically significantly higher compared to the other groups. On DOH, the MBL content in the livers of chickens in group II was statistically significantly higher than in groups I and III. At 7 days, the MBL content in the livers of chickens was statistically significantly lower in group III than in group IV. On DOH, the content MBL in the small intestine in group IV was statistically significantly higher than in groups I and II. On DOH, the MBL content in the large intestines of birds in group IV was statistically significantly higher than in group I. At 7 days, the content of MBL in the large intestine was statistically significantly higher in group IV than in group I. The content of MBL in the yolk sac in group I was statistically significantly lower than in groups II and III (Table 4).

### 3.3 Concentrations of immunoglobulins in the serum and tissues of chickens

On DOH, the concentration of IgA in the serum of chickens was statistically significantly higher in group IV than in the other groups. At 7 days, the serum IgA concentration in the group IV chickens was statistically significantly higher than in group I. On DOH, the IgA concentrations in the livers of chickens in groups II and IV were statistically significantly higher than in groups I and III. At 7 days, the IgA concentration in the livers of chickens from group II was statistically significantly higher than in the other groups. On DOH, a statistically significantly lower concentration of IgA was observed in the small intestines of birds from group II than in the other groups. At 7 days, the IgA concentration in the small intestine was statistically significantly lower in group IV than in the other groups. On DOH, the concentration of IgA in the large intestines of chickens from group II was statistically significantly higher than the other groups. At 7 days, the concentrations of IgA in the large intestines of birds in all supplemented groups were statistically significantly higher than in group I. Statistically significantly higher IgA concentrations were noted in the yolk sacs of birds in groups II and III compared to groups I and IV (Table 5).

On DOH, the serum IgY concentration of birds in group III was statistically significantly higher than the other groups. After 7 days, the serum IgY concentration in group I was statistically significantly higher than the other groups. On DOH, statistically significantly higher IgY concentrations were determined in the livers of chickens from groups III and IV compared to groups I and III. At 7 days, the IgY concentration in the livers of chickens from group I was statistically significantly lower than in the supplemented groups. On DOH, the IgY concentrations in the small intestines of birds from groups III and IV were statistically significantly higher than in groups I and II. After 7 days, the concentration of IgY in the small intestine in group IV was statistically significantly higher than in the other groups. On DOH, the IgY concentration in the large intestines of chickens from group III was statistically significantly lower than in the other groups. After 7 days, the IgY concentration in the large intestine of group III was statistically significantly lower than the other groups. A statistically significantly lower concentration of IgY was noted in the yolk sacs of birds from group II compared to groups I and III (Table 5).

On DOH, IgM concentrations in the serum of chickens from groups I and IV were statistically significantly lower than groups II and III. At 7 days, the IgM concentration in the serum of chickens from group III was statistically significantly lower than in the other groups. At 7 days, the IgM concentration in the livers of group II was statistically significantly lower than in the other groups. On DOH, the IgM concentration in the small intestines of birds from group I was statistically significantly lower than in groups II, III and IV. At 7 days, the IgM concentrations in the small intestines of chickens in all supplemented groups (II, III, and IV) were statistically significantly lower than in group I. In the large intestines analyzed on DOH, the IgM concentration in group III was statistically significantly lower than in the other groups. At 7 days, the IgM concentration in the large intestines of birds from group II was statistically significantly higher than in groups I, III, and IV. A statistically significantly lower IgM concentration was noted in the yolk sacs of birds from group IV compared to group I (Table 5).

### 3.4 IgA, IgM, and IgY mRNA expression ratios in the ileum

IgA, IgM, and IgY mRNA expression ratios in the ileum were statistically significantly higher at 7 days than on DOH. At 7 days, the IgA mRNA expression ratio was statistically significantly higher in group III than in the other groups, while the IgM mRNA expression ratio was statistically significantly higher in group IV than in group I (Table 6).

### 3.5 Levels of cytokines in chicken serum and tissues

On DOH, the level of IL-1β in the serum of chickens from group II was statistically significantly lower than in the other groups. At 7 days post-hatch, the serum level of IL-1β in chickens from group I was statistically significantly higher than in groups II, III and IV. Both on DOH and at 7 days, the IL-1β levels in the livers of chickens in group II were statistically significantly higher than in group IV. On DOH, the level of IL-1β in the small intestine was statistically significantly higher in group III than in the other groups. At 7 days, the IL-1β level in the small intestines of chickens in group IV was statistically significantly lower than in the other groups. On DOH, the IL-1β level in the large intestine was statistically significantly higher in group IV than in the other groups. At 7 days, the IL-1β level in the large intestine was statistically significantly lower in group I than in groups II, III and IV. The IL-1β level in the yolk sacs of chickens from group II was statistically significantly higher than in the other groups (Table 3).

On DOH, the level of IL-2 in the serum of chickens in group IV was statistically significantly higher than in group III. At 7 days, the serum IL-2 level in group IV was statistically significantly higher than in groups II and III. On DOH, the IL-2 level in the livers of chickens was statistically significantly higher in group I than in any of the supplemented groups. On DOH, the levels of IL-2 in the small intestines of chickens in groups III and IV were statistically significantly higher than in groups I and II. At 7 days, the IL-2 level in the small intestines of birds from group II was statistically significantly higher than in groups I and III. On DOH, the IL-2 level in the large intestines of chickens was statistically significantly higher in group IV than in the remaining groups (I–III). At 7 days, the IL-2 levels in the large intestines of chickens in groups III and IV were statistically significantly higher than in groups I and II. The IL-2 level in the yolk sacs of chickens from group III was statistically significantly lower than in groups I, II and IV (Table 3).

On DOH, statistically significantly higher levels of IL-6 were determined in the serum of chickens in groups I and IV compared to the remaining groups. On DOH, the IL-6 level in the livers of chickens from group II was statistically significantly higher than in the other groups (I, III, and IV). At 7 days, the level of IL-6 in the livers of chickens from group II was statistically significantly higher than in group III. On DOH, the IL-6 level in the small intestines of chickens from group III was statistically significantly higher than in the remaining groups. At 7 days, the IL-6 level in the small intestines of chickens from group IV was statistically significantly higher than in group III. On DOH, the IL-6 level in the large intestines of chickens from group IV was statistically significantly higher than in group III. At 7 days, the IL-6 level in the large intestines of chickens from group III was statistically significantly higher than in the other groups. The IL-6 level in the yolk sacs of chickens from group I was statistically significantly lower than in groups II, III, and IV (Table 3).

On DOH, the level of IL-8 in the serum of chickens from group IV was statistically significantly higher than in groups II and III. At 7 days, the serum IL-8 level in group IV was statistically significantly higher than in the other groups. On DOH, the IL-8 level in the livers of group II was statistically significantly higher than in the other groups. At 7 days, the IL-8 level in the livers of group III chickens was statistically significantly higher than in the other groups. On DOH, the IL-8 level in the small intestines of chickens from group IV was statistically significantly higher than in group II. At 7 days, the IL-8 level in the small intestines of chickens from group II was statistically significantly higher than in group III. At 7 days, the IL-8 level in the large intestines of chickens from group I was statistically significantly lower than in groups II, III, and IV. The IL-8 level in the yolk sac was statistically significantly higher in group IV than in the other groups (Table 3).

On DOH, the level of IL-10 in the serum of chickens from group III was statistically significantly lower than in groups I and IV. On DOH, IL-10 levels in the livers of chickens from groups III and IV were statistically significantly lower than in groups I and II. At 7 days, the IL-10 level in the livers of chickens from group III was statistically significantly lower than in group II. On DOH, the IL-10 level in the small intestines of chickens from group III was statistically significantly higher than in groups I and IV. At 7 days, the IL-10 level in the small intestines of chickens from group IV was statistically significantly higher than in group III. On DOH, the IL-10 level in the large intestines of chickens from group IV was statistically significantly higher than in the remaining groups. At 7 days, the IL-10 level in the large intestines of chickens from group III was statistically significantly higher than in groups I and IV (Table 3).

On DOH, the level of IL-12 in the serum of chickens from group I was statistically significantly higher than in group II. At 7 days, the serum IL-12 level in group IV was statistically significantly higher than in groups I and II. On DOH, the IL-12 levels in the livers of chickens from groups I and III were statistically significantly higher than in groups II and IV. At 7 days, the IL-12 level in the livers of chickens from group I was statistically significantly lower than in the supplemented groups (II, III, and IV). On DOH, the IL-12 level in the small intestines of chickens from group IV was statistically significantly higher than in the other groups. At 7 days post-hatch, the IL-12 levels in the small intestines of chickens from groups III and IV were statistically significantly higher than in groups I and II. On DOH, the IL-12 level in the large intestines of chickens from group IV was statistically significantly higher than in the other groups. At 7 days, the IL-12 levels in the large intestines of chickens from groups III and IV were statistically significantly higher than in groups I and II. The IL-12 level in the yolk sacs of chickens from group II was statistically significantly higher than in groups III and IV (Table 3).

On DOH, the level of TNF-α in the serum of chickens from group IV was statistically significantly higher than in group III. At 7 days post-hatch, the serum TNF-α level in group IV was statistically significantly higher than in the other groups. On DOH, its level in the liver of chickens from group I was statistically significantly higher than in groups III and IV. At 7 days, the TNF-α level in the liver was statistically significantly higher in group IV than in the other groups. On DOH, the TNF-α level in the small intestines of chickens from group I was statistically significantly higher than in groups II and III. At 7 days post-hatch, the TNF-α level in the large intestines of chickens from group III was statistically significantly higher than in group II. In turn, its level in the yolk sacs of chickens from group III was statistically significantly lower than in groups I and IV (Table 3).

On DOH, the level of IFN-γ in the serum of chickens from group IV was statistically significantly higher than in group III. At 7 days, the serum level of IFN-γ in group II was statistically significantly lower than in groups III and IV. On DOH, the IFN-γ level of the livers of chickens from group II was statistically significantly higher than in groups I, III, and IV. At 7 days, its level in group III was statistically significantly lower than in the other groups. On DOH, the IFN-γ level in the small intestines of birds from group III was statistically significantly lower than in groups I and IV. At 7 days, the IFN-γ level in the small intestines of chickens from group IV was statistically significantly lower than in groups I and II. On DOH, the IFN-γ level in the large intestines of chickens from group IV was statistically significantly higher than in group II. At 7 days, the IFN-γ level in the large intestines of chickens from group IV was statistically significantly higher than in the other groups. The IFN-γ level in the yolk sacs of chickens from group I was statistically significantly lower than in groups II, III, and IV (Table 3).

At 7 days, the serum level of TGF-β1 in group IV was statistically significantly higher than in groups I and II. On DOH, TGF-β1 levels in the livers of chickens from groups II and IV were statistically significantly lower than in groups I and III. At 7 days, statistically significantly higher levels of TGF-β1 were noted in the livers of chickens from group II compared to the remaining groups. On DOH, the TGF-β1 level in the small intestines of chickens in group I was statistically significantly lower than in the supplemented groups. On DOH, the TGF-β1 level in the large intestines of chickens from group IV was statistically significantly higher than in the other groups. At 7 days, the level of TGF-β1 in the large intestines of chickens from group I was statistically significantly lower than in groups II and III. The TGF-β1 level in the yolk sacs of chickens from group II was statistically significantly higher than in groups I and III (Table 3).

### 3.6 The relative weight of lymphatic organs in chickens

At 7 days, the relative weight of the spleens in group I was statistically significantly lower than in group II but did not differ statistically significantly from the other groups (III and IV) (Table 8).

## 4 Discussion

One of the major acute-phase proteins in poultry used as a biomarker of inflammation is SAA (Caliendo et al., 2013; Rhim et al., 2024). Its immunomodulatory activity plays an important role in homeostasis, contributing to the downregulation of inflammation (Urieli-Shoval et al., 2000). Chamanza et al. (1999) and Sevimli et al. (2008) showed that the SAA level increased due to the effect of the pro-inflammatory cytokines IL-1β, IL-6, and TNF-α on the body. Our study showed an increased SAA level in the small intestine tissue on DOH in the groups of birds receiving a multi-strain probiotic and Zn-Gly chelate. It is likely that changes in the composition of the microbiota cause the synthesis and release of SAA and the modulation of the local immune response in the intestine. It should be noted that these changes were not observed 7 days post-hatch. Moreover, at that time there were no changes in the SAA level in the serum and liver; hence, it is likely that the response was limited to tissues, without the development of generalized inflammation. However, the higher SAA concentration observed at 7 days post-hatch in the small and large intestines of birds receiving Zn *in ovo* in the form of glycine chelate may indicate that *in ovo* administration of zinc compounds may induce a stress response, such as due to the irritant effect of zinc on the intestinal epithelium. This leads to a local disturbance of homeostasis, limited to the intestinal tissue and manifested as an increased level not only of SAA (Alsemgeest et al., 1995) but of other proteins as well, such as haptoglobin, which was noted at high concentrations at 7 days post-hatch in chickens receiving Zn and the multi-strain probiotic *in ovo*.

It is worth noting the absence of differences between the control and experimental groups in the haptoglobin (Hp) concentration in the yolk sac, serum, and liver on DOH and at 7 days post-hatch. Increased Hp levels are usually noted during infections, inflammation, or tissue damage—for example, in infectious bronchitis virus (IBV), infectious bursal disease (IBD), retention disorders, and yolk sac infections (Nazifi et al., 2009; Nazifi et al., 2011; Mosleh et al., 2012). The absence of changes in Hp levels in the serum and yolk sac in our experiment allows us to exclude bacterial infections, systemic inflammations, or immunosuppressive diseases, whereas the changes in other APPs (SAA and AGP) should be ascribed to the effects of the compounds administered *in ovo*. The increase in the Hp concentration at 7 days post-hatch in the small and large intestines of birds receiving the multi-strain probiotic and Zn-Gly chelate may indicate successive multiplication of the gastrointestinal tract by the bacteria contained in the probiotic and environmental microbes stimulating local immune mechanisms. The high Hp concentration in the small intestine on DOH in the group of birds receiving the multi-strain probiotic indicates the implantation of the intestinal tissue by probiotic bacteria, which presumably stimulates immunocompetent cells of the GALT (gut-associated lymphoid tissue) system to synthesize Hp locally. In addition, rapidly dividing enterocytes together with probiotic bacteria stimulate metabolic processes in the intestines and the synthesis of proteins.

One of the most important APPs produced in the liver and secreted into the bloodstream is mannose-binding lectin (MBL) (Idowu et al., 2021). This protein, which is one of the collectins, plays an important role in non-specific immune response (Takahashi et al., 2006). In the present study, an increase in its concentration was noted on DOH in the yolk sacs in the group of birds receiving the multi-strain probiotic *in ovo*. Such a result may confirm the activation of the non-specific immune response already at the embryonic stage. In the liver, increased concentrations of this protein were shown in both the group receiving the multi-strain probiotic (group II) and the group administered the multi-strain probiotic and Zn-Gly chelate (group IV). These results demonstrate that probiotic bacteria administered *in ovo*, by colonizing the gastrointestinal tract of the developing bird before hatching, activate immune mechanisms which stimulate pathogen recognition processes and activate the specific immune response. Taking into account the results published so far, we can hypothesize that the preparations administered *in ovo* cause no imbalance in oxidative and antioxidant processes and that there are no ongoing inflammatory processes associated with oxidative stress, as evidenced by the low MBL levels in serum and tissues 7 days post hatching (Ciszewski et al., 2023b). It is notable that at 7 days post-hatch, the MBL concentration was also statistically significantly higher in the serum and large intestines of the birds receiving Zn-Gly chelate *in ovo* and in the large intestines of the birds administered the multi-strain probiotic and Zn-Gly chelate. This suggests that *in ovo* administration of Zn-Gly chelate can lead to a temporary stress response in newly hatched chicks due to the effect of highly bioavailable, easily absorbed zinc in organic form, accumulating in the tissues and organs. On the other hand, once accumulated in tissues, zinc may be involved in the temporary intensification of the immune response. It should also be emphasized that *in ovo* administration of a multi-strain probiotic and Zn-Gly chelate modulates the acute-phase response, particularly in the gastrointestinal tract. The changes observed are local and primarily affect the small intestine and yolk sac. The changes in SAA, Hp, and MBL concentrations observed in these tissues are likely transient, indicating that the compounds administered *in ovo* do not induce generalized inflammation or oxidative stress. At the same time, these data support the hypothesis that probiotics and zinc chelate exert an immunomodulatory effect, influencing the activation of innate immunity at the mucosal level. Another acute-phase protein typical of poultry is α1-acid glycoprotein (α-1-AGP), synthesized and secreted by hepatocytes. In the present study, no increase in AGP was shown in the liver either on DOH or at 7 days post-hatch. On the contrary, the AGP concentration was lower than in the control group on DOH and at 7 days post-hatch in the birds receiving the multi-strain probiotic and Zn-Gly chelate, respectively. Thus, we may speculate that the chickens did not suffer from any bacterial, viral, or systemic inflammation, which in natural conditions would lead to increased synthesis of AGP (Adler et al., 2001). The increased serum concentration of AGP in the chickens on DOH in the group receiving Zn-Gly chelate *in ovo* correlates with the increase in the serum levels of cytokines IL-1, IL-6, and TNF-α. These cytokines, IL-6 in particular, are considered to be factors that stimulate AGP synthesis in the liver and extrahepatic tissues (Tanaka et al., 2014). However, their concentrations determined in the liver tissue on DOH, except for IL-1, are relatively low. Thus, the temporary increase in the serum AGP level may be due to the redistribution of rapidly absorbed Zn to the tissues of the chicks. Taking into account the levels of other APPs (SAA, Hp, and Cp), we can hypothesize that the *in ovo* administration of non-specific immunogens, such as the non-pathogenic microbes contained in a multi-strain probiotic or highly bioavailable Zn in organic form, affects metabolic processes in the liver and immunocompetent cells regulating the strength of the immune response to exposure to them. This temporary stimulation is indicative of the anti-inflammatory and immunoregulatory effects of these proteins.

We also obtained interesting results regarding the concentrations of acute-phase proteins in the yolk sac on DOH. The concentrations of SAA, AGP, and Cp were highest in the group of the chickens receiving the multi-strain probiotic and Zn-Gly chelate *in ovo*. In the case of MBL, the highest concentration was noted in the group administered the multi-strain probiotic. The exact biological functions of these proteins in chickens and in developing embryos are not fully understood, despite the fact that their multi-faceted effects on the body have already been described (Gruys et al., 2005). The concentrations of acute-phase proteins in chickens in the period immediately after hatching, particularly in the yolk sac, have not been previously studied. APPs, such as AGP and SAA, may be produced locally in the oviduct or derive from the serum of laying hens and then enter the egg, including the yolk, which is the site of 16%–18% of proteins (Yadgary et al., 2010) that play an important role in the embryonic development of chicks. Approximately 119 proteins have thus far been identified in chicken egg yolks using 1-D SDS-PAGE, LC-MS/MS, and MS. The most abundant of these include serum albumin, vitellogenin (VTG) cleavage products, apovitellenins, immunoglobulin Y (IgY), α-fetoprotein, retinol-binding protein 4, and transthyretin (Zhu et al., 2020; Wu et al., 2022). This group of proteins also includes APPs, such as AGP, SAA, and CP, which in our study were present in high concentrations in the yolk sac immediately after hatching. These proteins can also be synthesized directly by the yolk sac tissues, influencing embryogenesis. It should be noted that APPs are also secreted by cells in which stress responses take place (Speelman et al., 2022). This occurs in the final period of chicken embryo development, in which changes in the lipid metabolism of the yolk—which is a source of energy for hormone synthesis, structural membranes, and cell replication and growth—trigger oxidative stress (Wong and Uni, 2021). These changes may cause a temporary increase in APP synthesis, which in our experiment led to an increase in the concentrations of AGP, SAA, and CP in the yolk sac on DOH. The changes in the levels of these proteins, however, may also have been due to *in ovo* administration of the multi-strain probiotic and Zn-Gly chelate. Their application may lead to changes in the microbiome of the developing chick, early bacterial habitation of the gastrointestinal tract, and increased absorption of highly bioavailable Zn from a chelate complex with glycine. These changes, in turn, result in an enhanced effect of Zn on the cells of the developing organism. This effect may have two different causes: temporary local inflammation resulting from the irritant effect of Zn on cells dividing rapidly during the growth and development of the chick or competition between probiotic and environmental microbes and those transferred to the egg by layer hens. Ceruloplasmin (Patel et al., 2002) is involved in antioxidant and cytoprotective defense, protecting tissues from free radical damage. A high concentration of this protein in the yolk sac is correlated with intensive metabolic processes (Wong and Uni, 2021) and prevents excessive tissue damage to the developing embryo. The Cp levels in the serum and tissues on DOH and at 7 days post-hatch in the experimental groups were comparable to the levels in the control group, or even lower. This indicates that preparations administered *in ovo* are safe for the chicken embryo and do not cause systemic inflammation, which could adversely affect its development. Furthermore, the low Cp levels confirm that the organism is not affected by stimuli inducing an inflammatory response, such as antigens of microbes, parasites, or endo- and exotoxins, which would be associated with inflammation and intensification of the acute-phase response (Georgieva et al., 2010).

Determinations of antibody levels in the serum and tissues of chickens and mRNA expression in the intestines provide interesting data. The results of the study showed that the IgY level in the yolk sac on DOH was equal to or lower than in the control group, which may suggest that the humoral response was not stimulated or that it was suppressed by these compounds. On the other hand, the concentration of this immunoglobulin in the serum and in the liver at 7 days post-hatch was statistically increased in the group administered the multi-strain probiotic and Zn-Gly chelate *in ovo*. This may suggest that IgYs specific to the chicken, present mainly in the serum, are involved in protection against environmental pathogens. Nevertheless, this is not a response to the substances administered *in ovo*, as evidenced by the low serum IgY level at 7 days post-hatch in the experimental groups, indicating that their *in ovo* application is safe. Moreover, in the groups of chicks which received the multi-strain probiotic together with Zn-Gly chelate (group III) or Zn-Gly chelate (group IV), the IgY level determined in the small intestine on DOH was higher than in the other groups. This may suggest that both IgY from the egg yolk and that synthesized by the chick, present in the small intestine, are involved in the control of intestinal pathogens, including those supplied with the microbes constitute the multi-strain probiotic. It seems that the increased IgY level in the groups receiving Zn in the form of highly bioavailable glycine chelate may be due to a stress response, such as that resulting from the irritant effect of zinc on the intestinal epithelium and the compensatory response of the organism, as well as to excessive stimulation of epithelial enterocytes and stimulation of local immune mechanisms, leading to increased B cell proliferation and antibody synthesis induced by the excessive intake of zinc in the chelated form. This observation is confirmed by the high IgY level in the small intestine tissue 7 days post-hatch in the group administered Zn-Gly chelate *in ovo*. It should be borne in mind, however, that the high bioavailability of Zn may induce a local inflammatory process (Bonaventura et al., 2015), and a high IgY concentration is aimed at protecting the intestinal mucosa against invasion by intestinal pathogens. Moreover, no differences were shown in IgY mRNA expression in the intestinal tissue between groups on DOH or at 7 days post-hatch, which confirms that the multi-strain probiotic and Zn-Gly chelate applied *in ovo* do not induce a systemic inflammatory response; their effect is limited to the stimulation of the mucosa-associated lymphoid tissue (MALT) system.

In the present study, the IgA level determined on DOH was high in the yolk sac in the groups of birds receiving the multi-strain probiotic *in ovo*. This increase in IgA may have been due to the transfer of these antibodies from the egg albumen to the yolk and then to the yolk sac or from the embryonic intestine to the yolk sac since day 14 of incubation (Ismiraj et al., 2019). Serum IgA levels on DOH and at 7 days post-hatch were higher in the group of birds receiving Zn-Gly chelate *in ovo*, whereas IgA levels in the liver were increased on DOH in the groups of birds administered the multi-strain probiotic and Zn-Gly chelate and at 7 days post-hatch in the group receiving the multi-strain probiotic. This may be indicative of the stimulation of humoral immune mechanisms by the multi-strain probiotic and Zn through the stimulation of local immunocompetent cells, including B lymphocytes that produce antibodies. Kaspers et al. (1991) excluded the embryonic synthesis of IgA and IgM and showed that their production begins 2–4 days post-hatch in the case of IgM and 6–13 days post-hatch in the case of IgA. Contrasting findings were presented by Thorbecke et al. (1968), who showed that bursa cells in 18-day embryos were already capable of synthesizing IgM. Nevertheless, *in ovo* supplementation with a multi-strain probiotic and Zn-Gly chelate may also stimulate local antibody synthesis.

Interesting results were obtained in the present study for the IgA and IgM levels in the small intestine tissue. In the case of IgA, its concentration on DOH was lower in the experimental groups than in the control group, while the opposite was true for IgM on the same day. At 7 days post-hatch, IgA and IgM levels in the small intestine tissue were lower or comparable to those found in the control group. A high IgA concentration, mainly in the intestines, is usually associated with damage to the intestinal barrier and exposure to numerous antigens (Fan et al., 2024). The low concentration of this immunoglobulin determined in the intestinal tissue in the present study indicates that the intestinal barrier was not damaged. The potential change in the composition of the microbiome due to *in ovo* supplementation with the multi-strain probiotic and the supply of numerous antigens to the body also did not lead to the development of an inflammatory response. This status may be confirmed by the suppressed synthesis of pro-inflammatory cytokines IFN-γ and TNF-α and the enhanced synthesis of anti-inflammatory cytokines IL-10 in the small intestine tissue. The increased IgM level in the intestinal tissue on DOH in the experimental groups is most likely the effect of the intensification of the GALT system response to antigenic stimulation during the rearrangement of the intestinal microbiome.

Changes in mRNA expression of these immunoglobulins were not observed until 7 days post-hatch. In each of the experimental groups, mRNA expression was higher than in control. In the case of IgA, it was highest in the group of birds receiving the multi-strain probiotic and Zn-Gly chelate, while mRNA expression of IgM was highest in the group administered the Zn-Gly chelate. It should be noted that probiotics can increase the number of cells producing IgA in the intestinal mucosa and lamina propria (Ohland and MacNaughton, 2010; Levkut et al., 2014). The increase in the mRNA expression of IgA and IgM indicates that the application of a mixture of beneficial bacteria in a multi-strain probiotic stimulates the proliferation and activity of B cells within the intestinal mucosa, which leads to the enhanced local production of immunoglobulins and thus protects the intestinal epithelium against invasion by pathogenic microbes, thereby ensuring homeostasis in the intestines.

Improvement in growth parameters is influenced by numerous factors, including increased villus height and crypt depth, lower FCR, and the intensification of gluconeogenesis processes in chicks and adult birds (El-Moneim et al., 2020; Das et al., 2021). It should be stressed that the administration of the multi-strain probiotic and Zn-Gly chelate did not significantly affect BWG or FCR (0–42 days) in the chickens receiving them *in ovo*, although in group II, which received only the multi-strain probiotic, BWG (0–42 days) was lower than in the control group and experimental groups III and IV. Similar results were reported by Majidi-Mosleh et al. (2016); Majidi-Mosleh et al. (2017), who noted no changes in the growth parameters of chicks following *in ovo* administration of strains of *Bacillus subtilis*, *Enterococcus faecium* and *Pediococcus acidilactici*. They did observe, however, that the probiotics had a beneficial effect on the expression of the mucin gene and microbiota proliferation in the jejunum, and they recommended *in vivo* supplementation with probiotics in addition to *in ovo* technology. Contrasting findings were reported by Ravichandran et al. (2018), who showed that *in ovo* administration of various concentrations of *Lactobacillus acidophilus* increased body weight in broilers at 6 weeks of age. Shehata et al. (2022) also showed that the *in ovo* administration of *B. subtilis* increased BW, FI and FCR compared to the control group. According to these studies, this effect should be linked to improved intestinal function, an increase in the population of beneficial bacteria, a reduction in the population of harmful bacteria, regulation of the expression of mucin-2 genes, vascular endothelial growth factor (VEGF), sodium/glucose cotransporter 1 (SGLT1), interleukin 2 (IL-2) in the intestines, and a positive effect on their morphology. Discrepant results have also been obtained regarding the *in ovo* application of zinc. Kim and Kang (2022) showed no significant effect of zinc administered *in ovo* on body weight gain, feed intake, or FCR in the first 21 days of the life of broilers. The effects of this supplementation did not appear until 35 days of age, when a higher body weight was noted in broilers administered zinc *in ovo* and additionally in their feed. In turn, Hamza et al. (2022) demonstrated that *in ovo* administration of ZnSO_4_ or ZnO-NPs increased the body weight of chickens at 21 and 35 days of age. These differences in the cited findings may be due to the effect of numerous internal and external factors affecting growth parameters in the case of *in ovo* technology: the dosage of supplements, the composition of the probiotic mixture, the day of administration, the *in ovo* technique used, the site of administration, the origin of the hatching eggs, the strain/line/breed of bird, egg storage conditions, and differences in the feed administered from hatching to slaughter at 42 days. It should be noted that in our experiment, the probiotic microorganisms and Zn-Gly chelate administered *in ovo* had a strong impact on the health of the chicks, which we also demonstrated in our previous studies (Ciszewski et al., 2023a; b). It is likely that zinc had no tangible effect on the growth rate because the birds’ requirement for this element and other nutrients was fully met in the eggs from which the chicks hatched. This was the interpretation used by Kop-Bozbay and Ocak (2015), who observed that *in ovo* supplementation with amino acids had no effect on growth parameters when they used hatching eggs with an optimal nutrient content.

In the present study, BWG in the first period of life (0–10 days) was higher in the group receiving the multi-strain probiotic and Zn-Gly chelate *in ovo* (group III) and the group receiving Zn-Gly chelate (group IV) than in the control group, but these values were not statistically significant. Similar observations were made by Khaligh et al. (2019), who found that birds supplemented *in ovo* with *Lactobacillus salivarius* and *L. plantarum* achieved higher body weight in the first 10 days post-hatching. This was most likely due to the intensified absorption of nutrients by the yolk sac after hatching, which stabilized with age. Duan et al. (2021) also showed that *in ovo* application of a synbiotic increased BWG and FI between days 7 and 21 post-hatch, which indicates the promotion of early growth in chickens. In the present study, the temporary increase in BWG in the first days post-hatch may be due to the intensification of liver metabolism and nutrient absorption from the yolk sac. The results obtained indicate that *in ovo* supplements do not affect production efficiency and, at the same time, do not stimulate growth under optimal feeding conditions. Interesting data were also obtained concerning the weight of the lymphatic organs. The development and growth of the bursa of Fabricius, thymus, and spleen were not affected by *in ovo* supplementation with either the multi-strain probiotic or Zn-Gly chelate in the period immediately after hatching. At 7 days post-hatch, statistically significant differences were shown only for the weight of the spleen, which was greater in all experimental groups than in control, and highest in the groups of birds receiving the multi-strain probiotic. Previous studies have indicated that supplementation with Zn, such as in the form of nanoparticles (ZONPs), increases the relative weight of immune organs like the thymus, bursa of Fabricius, and spleen (El-Haliem et al., 2020). A similar effect has been reported following *in ovo* and *in vivo* administration of probiotics (Pender et al., 2017). Duan et al. (2021) also showed that *in ovo* administration of synbiotics led to an increase in immune organ indices, especially for the thymus and spleen, which improved the overall health of chickens and boosted their resistance to pathogens. It should be emphasized that the gastrointestinal microbiota and its metabolites act closely with GALT cells in chickens and play an important role in the development of local and systemic immune mechanisms. The early development of beneficial gut bacteria through *in ovo* supplementation—for example, with probiotics—can therefore enhance the systemic response in chickens. The increase in the weight of the spleen at 7 days post-hatch is due to the migration and early development of probiotic microbiota in the gastrointestinal tract following *in ovo* administration; however, stimulation by environmental strains is also likely. The microbiota modulated *in ovo* activated immunocompetent cells, mainly of the intestinal lymphoid epithelium and the GALT system, leading to an increase in antigen migration to the spleen. Antigen burden results in enhanced immune reactivity, leading to an increase in the size of the spleen—important for the health and immunity of birds (Fairbrother et al., 2004). The increase in spleen mass may therefore indicate increased recruitment of lymphocytes and phagocytic activity—phenomena typical of the activation of non-specific and adaptive immunity. Interesting results were obtained regarding the cytokine concentrations in the yolk sac. The groups receiving the multi-strain probiotic (group II) *in ovo* had higher levels of IL-1β, IL-6, IL-8, IL-12, TNF-α, IFN-γ, and TGF-β1. At the same time, administration of the multi-strain probiotic and Zn-Gly chelate increased the concentrations of IL-6, IL-8 and IFN-γ. It is notable that the high expression of IFN-γ, IL-4, IL-10, and IL-18 is already found in the lymphatic organs of chickens, including the spleen, on day 12 of incubation, and persists up to 7 days post-hatch (Abdul-Careem et al., 2007). The results of the present study also clearly indicate the dominance of a pro-inflammatory cytokine profile in the yolk sac. The increased synthesis of Th1 pro-inflammatory cytokines in the yolk sac, like IL-1β, may be due to the activation of the intracellular NF-κB signaling pathway under the influence of antigenic stimulation (Rehman et al., 2021) by the probiotic strains contained in the multi-strain probiotic. Similarly, in the groups receiving Zn, the activation of pro-inflammatory pathways during embryonic development may be linked to the intensification of metabolic processes which use this element to build structural elements of the body, including cells involved in the immune response (Klasing, 2007).

In the present study, in the groups of birds receiving the multi-strain probiotic and Zn-Gly chelate *in ovo*, the concentrations of the pro-inflammatory cytokines IL-2, IL-6, IL-8, IL-12, TNF-α, and IFN-γ determined in the serum and liver tissue immediately after hatching were low or comparable to the levels found in the control group. Similar observations were reported by Alizadeh et al. (2020), who showed that *in ovo* inoculation with multi-strain lactobacilli diminished the expression of genes of pro-inflammatory cytokines in the cecal tonsils, which indicates the anti-inflammatory activity of these bacteria in the intestine. The multi-strain probiotic administered *in ovo* in our experiment also contains *Lactobacillus* strains. The fact that this probiotic reduced the concentration of some cytokines, such as IL-2, which is synthesized by activated T lymphocytes involved in the proliferation and activation of Th (helper) and Tc (cytotoxic) cells, is indicative of the immunomodulatory properties of bacteria in naive chickens that have not been exposed to viral and bacterial infections. The low IL-6 and IL-8 levels resulting from the *in ovo* modulation of the microbiota may prove that probiotic bacteria are involved in maintaining immune homeostasis, preventing excessive development of the inflammatory response. At 7 days post-hatch, on the other hand, we showed an increase in the concentrations of pro-inflammatory cytokines in the liver (IL-1β, IL-2, IL-8, IL-12, and TNF-α) and serum (IL-6, IL-12, and IFN-γ) in the groups administered the multi-strain probiotic and Zn-Gly chelate *in ovo*. This increase should be interpreted as an element of the body’s physiological adaptation to environmental antigens and not as a pathological inflammatory state. Alizadeh et al. (2021) also showed upregulation of the expression of interferon (IFN)-α, IFN-β, IL-8, IL-13, and IL-18 in the spleen and of IFN-γ, IL-2, IL-6, IL-8, IL-12, and IL-18 in the bursa of Fabricius in 10-day-old chicks administered a lactobacilli cocktail *in ovo*. The results of the present study suggest that *in ovo* application of a multi-strain probiotic and Zn-Gly chelate modulates the immune response, maintains balance between the synthesis of Th1 and Th2 cytokines, inhibits inflammatory processes, and stimulates immune system development. This hypothesis may be supported by the high concentration of IL-6, which drives the differentiation of T lymphocytes toward the Th2 phenotype and stimulates the secretion of anti-inflammatory cytokines, leading to the suppression of an excessive immune response. It is also worth citing the results obtained for the serum concentrations of IL-8 and IFN-γ, which directly after hatching were lower in the groups of birds receiving the multi-strain probiotic and Zn-Gly chelate than in the control. The inhibition of inflammatory functions shown in the experiment may be due to the suppression of the immune response following the use of the supplements, which may have impaired the activation of T cells essential to an effective immune response following post-hatch vaccination (Haq et al., 2011). On the other hand, this phenomenon can be treated as a temporary impairment in the synthesis of proteins due to metabolic changes taking place during hatching (Meijer et al., 2024). Nevertheless, it is undeniable that the changes in the concentrations of these cytokines are closely linked to a chick’s ontogenetic development.

Opal and DePalo (2000) showed a reduction in the level of IL-10 in the serum and an increase in its level in the liver after hatching in groups receiving Zn-Gly chelate *in ovo*. The higher IL-10 level in the liver may be due to the activation of B lymphocytes by zinc, directly or via other cytokines released from immunocompetent cells, which enhances humoral immune mechanisms. The activation of these processes is also evidenced by the increased concentrations of immunoglobulins IgA and IgY in the liver in the experimental groups. The low serum level of IL-8 in the groups administered the multi-strain probiotic and Zn-Gly chelate *in ovo* coupled with a low IFN-γ level may indicate the absence of inflammation at this time or the inhibition of the Th2 immune response. Similarly, at 7 days post-hatch, the lack of differences in the IL-10 concentration in the serum and its decrease in the liver, especially in the group receiving the multi-strain probiotic and Zn-Gly chelate *in ovo*, coupled with high IFN-γ and TNF-α levels, point to the inhibition of the humoral response.

Comprehensive analysis of cytokine concentrations in the tissues of the small and large intestine and in the liver indicate tissue-specific determinants regulating cytokine synthesis that are dependent on tissue and organ development as the chick grows older as well as on antigenic stimulation. It should be noted, however, that no group of cytokines was shown to be dominant, and the results indicate that a balance was maintained in the production of type Th1 and Th2 cytokines. Differences in the concentrations of cytokines in the experimental groups between DOH and 7 days post-hatch can be presumed to depend on the age of the chicks and to be regulated not only by internal factors but also by the degree of maturity of the immune system. It is worth noting that on DOH, the mRNA expression of IgM in the intestines was low, which may have been due to the activation of a pro-inflammatory response weakening the adaptive mechanisms of the immune response. The simultaneous high concentration of anti-inflammatory IL-10 in the tissue of the small and large intestine following *in ovo* administration of the multi-strain probiotic and Zn-Gly chelate suggests the existence of mechanisms balancing the inflammatory response profile, leading to a shift from a cellular to a humoral response (Giansanti et al., 2006). By striving to maintain the Th1/Th2 balance despite the antigen burden associated with *in ovo* supplementation, the developing immune system effectively maintains systemic homeostasis.

Ciszewski et al. (2023a) demonstrated that *in ovo* administration of multi-strain probiotics and Zn-Gly chelate significantly reduced hatchability, suggesting that *in ovo* injection of the two agents negatively impacts embryo survival. It is possible that the *in ovo* administration technique causes accidental damage to egg structures or microbiological contamination; the supplements may be toxic or disrupt homeostasis and impair embryo development. In our experiment, the synergistic use of the two compounds could have affected the pH and osmotic pressure of the embryo, triggering an immune response, translating into reduced hatchability. Therefore, it is necessary to optimize the doses of the *in ovo* tested supplements to minimize hatching disruptions. However, the preparations used in the experiment did not affect the viability of the chicks after hatching. The lack of significant differences in body weight after hatching between the control and experimental groups suggests that *in ovo* supplementation with the tested preparations did not inhibit embryonic growth. During the initial period of rearing, no increased growth of “*in ovo* feeding” chicks as was observed. Post-hatch mortality also remained low. Only the group receiving the multi-strain probiotic showed higher mortality in the first days of life, which could have been the result of a microbial imbalance (dysbiosis) or an abnormal immune response (inflammatory response). The few scientific reports regarding in *ovo* probiotic injection indicate the safety of such procedures (Shehata et al., 2024). Our results encourage the search for alternative methods of supplementing these compounds.

## 5 Conclusions

*In ovo* supplementation with a multi-strain probiotic and zinc-glycine (Zn-Gly) chelate stimulates immunocompetent cells, metabolic processes, and the synthesis of acute-phase proteins in the liver. It also enhances immunoglobulin mRNA synthesis, thereby modulating the immune response in broiler chickens. The coupled use of a multi-strain probiotic and Zn-Gly chelate results in increased expression of anti-inflammatory cytokines (IL-10) while simultaneously reducing the levels of pro-inflammatory cytokines (IFN-γ and TNF-α), indicating a lack of excessive inflammatory response, maintenance of Th1/Th2 balance, and homeostasis in the body. Elevated SAA levels in the intestinal tissue and increased expression of IgA and IgM mRNA indicate the activation of local mucosal immunity. An increase in spleen weight was observed despite the lack of significant differences in growth parameters (BWG and FCR) and in the weight of the thymus and bursa of Fabricius, which is indicative of increased immune reactivity and impact on chick immunity post hatching. Although the synergistic use of both supplements *in ovo* may reduce hatchability, this type of supplementation is becoming a promising tool for strengthening immunity in newly hatched chicks, which may have practical implications for preventive healthcare in flocks under intensive poultry production conditions.

## Data Availability

The original contributions presented in the study are included in the article/Supplementary Material, further inquiries can be directed to the corresponding author.
