# Supplementary material for: Influence of a multi-strain probiotic and zinc-glycine chelate, administered in ovo, on immune response in newly hatched chicks
Source: Front Physiol. 2025 Sep 22;16:1646143. doi: 10.3389/fphys.2025.1646143 (PMC12497791; doi:10.3389/fphys.2025.1646143)
Supplement: Supplementary file 1 [file Table1.docx]

Supplementary Material

# Supplementary Table S1.

# Chick mortality during the experiment.

| **Group** | **Starter** | **Grower** | **Finisher** | **Total (percent mortality)** |
| --- | --- | --- | --- | --- |
| I | 1 | 0 | 0 | 1 (0.67%) |
| II | 3 | 2 | 0 | 5 (3.33%) |
| III | 1 | 0 | 1 | 2 (1.33%) |
| IV | 2 | 0 | 0 | 2 (1.33%) |
| P-value |  |  |  | 0.362 |
